# Clinical Implementation of Proton Therapy Using Pencil-Beam Scanning Delivery Combined With Static Apertures

**DOI:** 10.3389/fonc.2021.599018

**Published:** 2021-05-12

**Authors:** Christian Bäumer, Sandija Plaude, Dalia Ahmad Khalil, Dirk Geismar, Paul-Heinz Kramer, Kevin Kröninger, Christian Nitsch, Jörg Wulff, Beate Timmermann

**Affiliations:** ^1^ West German Proton Therapy Centre Essen, Essen, Germany; ^2^ West German Cancer Center (WTZ), University Hospital Essen, Essen, Germany; ^3^ German Cancer Consortium (DKTK), Heidelberg, Germany; ^4^ Faculty of Physics, TU Dortmund University, Dortmund, Germany; ^5^ Department of Particle Therapy, University Hospital Essen, Essen, Germany

**Keywords:** proton ****therapy, pencil-beam scanning, aperture, quality assurance, radiation protection, brain tumors, ocular tumors

## Abstract

Proton therapy makes use of the favorable depth-dose distribution with its characteristic Bragg peak to spare normal tissue distal of the target volume. A steep dose gradient would be desired in lateral dimensions, too. The widespread spot scanning delivery technique is based, however, on pencil-beams with in-air spot full-widths-at-half-maximum of typically 1 cm or more. This hampers the sparing of organs-at-risk if small-scale structures adjacent to the target volume are concerned. The trimming of spot scanning fields with collimating apertures constitutes a simple measure to increase the transversal dose gradient. The current study describes the clinical implementation of brass apertures in conjunction with the pencil-beam scanning delivery mode at a horizontal, clinical treatment head based on commercial hardware and software components. Furthermore, clinical cases, which comprised craniopharyngiomas, re-irradiations and ocular tumors, were evaluated. The dosimetric benefits of 31 treatment plans using apertures were compared to the corresponding plans without aperture. Furthermore, an overview of the radiation protection aspects is given. Regarding the results, robust optimization considering range and setup uncertainties was combined with apertures. The treatment plan optimizations followed a single-field uniform dose or a restricted multi-field optimization approach. Robustness evaluation was expanded to account for possible deviations of the center of the pencil-beam delivery and the mechanical center of the aperture holder. Supplementary apertures improved the conformity index on average by 15.3%. The volume of the dose gradient surrounding the PTV (evaluated between 80 and 20% dose levels) was decreased on average by 17.6%. The mean dose of the hippocampi could be reduced on average by 2.9 GyRBE. In particular cases the apertures facilitated a sparing of an organ-at-risk, e.g. the eye lens or the brainstem. For six craniopharyngioma cases the inclusion of apertures led to a reduction of the mean dose of 1.5 GyRBE (13%) for the brain and 3.1 GyRBE (16%) for the hippocampi.

## Introduction

The depth dose characteristics with its distinct Bragg Peak facilitate a conformation advantage of proton beam treatment fields over hard X-ray fields. In lateral directions, however, the lateral dose fall-off limits the options to cover the target volume while keeping the dose to organs at risk low. This holds particularly true for the pencil-beam scanning delivery method (PBS), which has gained importance over passive delivery techniques with collimating apertures in the last few years. The conjunction of pencil-beam scanning with apertures constitutes a hybrid of PBS and passive beam delivery facilitating a good lateral dose fall-off (1–6). PBS-with-apertures can be technically realized through static apertures, multi-leaf collimators or dynamic collimators, which are synchronized with the PBS delivery (7–10). The concerns toward PBS with static apertures are similar to those toward passive delivery techniques: extra costs of the aperture production, extra time for the radiation therapy technologists to exchange beam shaping devices during a treatment session, less flexibility for plan adaptation, and radiation protection issues regarding handling and storage. The advantage of PBS with static apertures is the possibility to realize small air gaps in clinical treatment plans, which is beneficial for the lateral dose gradient (5, 11). Previously, multi-leaf collimators featured large air gaps (11). Recently, dynamic adaptive collimators, which enable small air gaps, were clinically introduced (9, 10). These dynamic collimators are necessary to compensate for wide PBS spots from a gantry-mounted cyclotron. Auxiliary beam-shaping hardware for proton PBS is used mainly for targets at shallow to medium depths, because the contribution of scattering in the patient dominates the lateral dose gradient at large radiological depths. The technical design of the proton treatment machine dictates the options for the clinical user to employ PBS-with-apertures. For instance, in the current study the mechanics supporting PBS with static apertures is available, because a multi-modal proton nozzle supporting active and passive delivery modes is used. Alternatively, dedicated PBS nozzles can be equipped with an extra holder for apertures.

The current work gives an account of the implementation of PBS with static, field-specific apertures in our proton therapy facility. The hardware and software set-up and the quality assurance (QA) are described in Section 2. This section also contains a description of the treatment planning techniques together with the design of the in-silico study, which compares the dose distribution of clinically applied treatment plans using brass apertures with the respective plans without supplementary apertures. Section 3 presents the results of the *in silico* plan comparison. We considered pediatric patients, re-irradiations, and small target volumes including eye tumors and stereotactical treatments as potential cases, which would benefit from the addition of apertures to PBS treatment fields. Section 4 discusses the possible clinical benefits of the supplement of PBS fields with apertures and the possible radiation protection issues.

## Materials and Methods

### Proton Therapy Equipment

The delivery of proton fields was facilitated by the ProteusPlus therapy machine (IBA, Lovain-La-Neuve, Belgium) operated in spot-by-spot PBS mode. Proton acceleration and beam delivery are controlled by the treatment control system (TCS). Protons are accelerated in the isochronous cyclotron to the maximum kinetic energy of about 228 MeV. The energy can be reduced continuously down to 100 MeV downstream of the cyclotron with a wheel mounted wedge. The beam is cleaned up with analyzing magnets and slits and subsequently lead to a fixed-beam treatment room with horizontally mounted treatment head. **Table 1** gives an overview of the spot sizes, which are typical of the fields applied in the frame of the current study. The treatment head used for the current study was the so-called “universal nozzle” which supports single scattering, double scattering, uniform scanning, and PBS. It contains thin transmission monitor ionization chambers and a snout. The snout of type “Snout180” can be moved in beam direction to optimize the air gap. It provides slots for two brass apertures and a holder for range shifters (PBS) or range compensators. The patients were positioned (30 cases in supine position) on a PatLog air plate couch which was mounted to the patient positioning system (PPS). The PPS can be translated in three directions and rotated in the horizontal plane by ±180°. Additionally, angular corrections in pitch and roll directions are feasible up to ±3°. The patient set-up was verified with the X-ray based patient positioning and verification system (PPVS) which comprised three orthogonal X-ray panels. Generally, the X-ray imaging of the PPVS constitutes the geometrical reference for the isocenter position. The X-ray panel-A images in beam direction. It is used for quality assurance of the spot position (*Quality Assurance*).

**Table 1:** Pencil-beam scanning in-air spot characteristics of the treatment plans of the current study.

Energy	σ	Comment
(MeV)	(mm)	
100	8.1	minimum energy among all cases
110	7.4	median of the lowest energy of all cases
130	6.5	median energy of all cases
150	5.3	median of the highest energy of all cases
170	4.6	maximum energy among all cases

Fields were applied with the IBA universal nozzle of the fixed-beam treatment room of WPE. σ refers to the standard deviation derived from a fit of a Gaussian distribution to the lateral spot profile. Proton energy values of the layers were rounded, because spot characteristics were measured in the isocenter plane in steps of 10 MeV in the clinical commissioning.

### Aperture Production and Use

The brass material was composed of 58% copper, 39% zinc and 3% lead. In our milling-machine shop (MMS) non-divergent brass apertures with a thickness of 3.3 cm were fabricated with a computer numerical control milling machine (Leadwell/Taiwan). The milling head diameter was 1 cm. Aiming for an efficient production and treatment workflow, the actual number of apertures used was optimized per treatment field. It depended on the residual range R80 (in water) at the exit of the snout, i.e. it accounted for the energy degradation of an optional range shifter. In any case a field-specific aperture was mounted in the downstream slot (see *Proton Therapy Equipment*). If R80 exceeded 15.5 cm, a second field-specific aperture was mounted in the upstream slot. Otherwise, an open ring aperture was inserted in the upstream slot.

### Treatment Planning and Dosimetric Analysis

A total of 31 patients (average age of 16 y, median age of 10 y, 21 patients <18y) were planned and treated in a fixed gantry room using PBS-with-apertures. The target volumes (5.4 to 229.0 cm^3^, median 33 cm^3^) were located in the skull. Cases were diagnosed, e.g., with craniopharyngimonas (n = 6) and ependymomas (n = 4). Some cases (n = 5) were retreatments. **Table 2** provides more detailed information.

**Table 2:** Overview of all cases which were treated with PBS-with-apertures and which were evaluated in the current study.

Localization	Diagnosis	Number of patients/retreatments	Average age (years)	V_PTV_ (cm^3^)
Orbital tumors	Retinoblastoma	3	3	9.0
	Embryonal Rhabdomyosarcoma	1	12	16.2
	Optic nerve Glioma	1	14	113.8
	Choroidal Melanoma	1	51	8.6
	Choroidal Hemangioma	1	20	12.0
Base of skull tumors	Optic Posterior Pathway Glioma	1	8	67.0
	Brainstem Glioma	1	2	31.5
Intracranial tumors	Clivuschordoma	1	58	20.9
				
(infratentorial)	Ependymoma	6/2	9	22.5
	Atypical Teratoid Rhabdoid tum.	1/1	6	30.8
(supratentorial, midline)	Craniopharyngioma	7/1	12	97.6
	Neurocytoma	1	20	27.0
	Germ Cell tumor	3/1	8	52.4
	Astrocytoma	1	37	38.7
(supratentorial, unilateral)	Astrocytoma	2	42	173.7

For patient groups the age and the average volume of the PTV (V_PTV_) are listed.

The treatment planning system (TPS) RayStation (versions 6 and 7; RaySearch Laboratories, Stockholm/Sweden) was used (12). Dose distributions were simulated with the Monte Carlo (MC) dose engine (13), which transported primary and secondary protons with a Class II method. Secondary deuterons and alpha particles were tracked in the continuous slowing down approximation, i.e. their energy loss was accounted for while scattering, straggling and nuclear interactions were disregarded. The source of primary protons was located upstream of the beam shaping devices. Thus, the MC transport accounted for effects like edge scattering at the inner aperture surface and large-angle scattering in the range shifter. Secondary neutral particles were not simulated in the MC dose engine.

**Figure 1:**
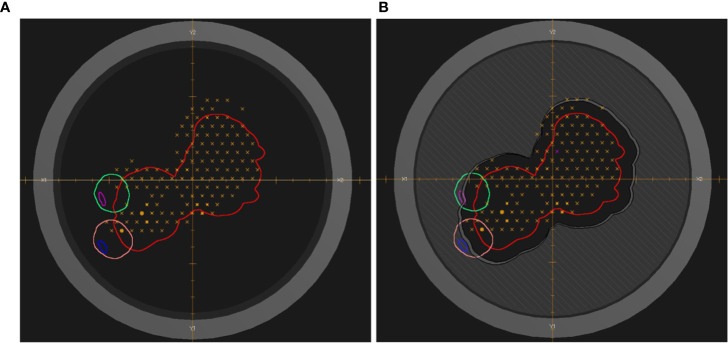
Beams-eye view of a treatment field of the uncollimated plan **(A)** and the treatment plan with apertures **(B)**. The red contour indicates the PTV. The pink (blue) and green (magenta) contours indicate the eye (lens). The dark gray hatched area visualizes the brass of the aperture. The dark gray annular ring represents the snout holder. Orange crosses and circles indicate the centers of individual pencil-beam scanning spots of one of the energy layers. The dose distribution of the treatment plan is shown in **Figure 6**.

In the initial planning phase the RayStation optimization module was used to customize the spot fluences of the treatment plan. Robust optimizations were performed with an isotropic 3 mm isocenter shift and 3.5% density uncertainty. Treatment plan optimizations started with a single-field uniform dose strategy. This proved to be adequate for most cases. In other cases a restricted multi-field optimization with dose modulations up to ±20% per field was realized. The resulting plan, which fulfilled most of the clinically relevant dose requirements, could already be applied clinically. In the scope of the current study, it served as a reference for a typical PBS treatment plan. It will be called “uncollimated plan” in the following. Starting with the uncollimated plan, apertures were inserted for all fields specifying a lateral margin for the PTV coverage and blocking of adjacent organs at risk (OAR), if necessary. The margin accounts for the dose fall-off between the 50% and >90% isodose lines with the 50% isodose line coinciding with the projected aperture edge for a uniform field and a homogenous phantom. It was, thus, adapted for each individual field. The spot positions exceeded the aperture boundary by typically 5 mm in the beams-eye-view isocenter plane (“overscan”). **Figure 1** shows an example of the beams-eye view of an uncollimated treatment field compared to its collimated implementation.

The number of fields and their arrangement were chosen depending of the localization of the target volume. For the treatment of ocular tumors on average 1.6 fields were used, since for the most of these cases the target volume is superficial, small and with a simple geometry. Centrally located target volumes like craniopharyngiomas and tumors of the ventricle system were treated with three fields, i.e. two lateral and one vertex field, to decrease the dose to temporal lobes and hippocampi. For the target volumes in the skull base and those located more asymmetrically against the middle line of the brain, two fields were chosen to avoid treating from contralateral side. The air gap was chosen as small as possible to reduce the lateral penumbra while avoiding the possible collision. For fields delivering dose to shallow radiological depths range shifters were used. The range shifter was placed downstream of the aperture. The thinnest range shifter was chosen from a set of three range shifters for each individual field.

The following quantities were used to evaluate the dosimetric performance of PBS-with-apertures:

- ΔV_20-80%_ : quantifies the dose reduction in the dose gradient around the PTV. Maes et al. (5) compared the distance between the 20 and 80% isodose line of PBS fields with and without apertures, in order to characterize the lateral penumbra. Since the patient anatomy is not homogeneous [as in Ref. (5)] and the geometry of targets is quite complicated, a difference of volume between 80 and 20% isodose lines was analyzed with operations on regions-of-interest in RayStation. ΔV_20-80%_ combines the 80–20% fall-off distance of Ref. (5) with the 10 mm ring surrounding the PTV of Refs. (8, 14).
- CI 50% (the conformity index of the 50% dose level of the prescription dose) was used to quantify improvements of the dose conformity (14). CI 50% was computed in RayStation as the ratio between the volume of region of interest (ROI) covered by 50% isodose line and the volume of the PTV.

For the three retinoblastoma cases (**Table 2**) the 20 and 50% isodose lines would partly lie in a volume outside the body and external contours. RayStation forces the corresponding regions of these isodose lines to the external contour. The dose statistics for the considered OARs like optic nerve, brainstem, thalamus, and hippocampus were assessed with RayStation using a dose computation on a 2 mm grid. Accounting for the variation of clinical goals, location, shape and volume of the target as well as the dose concept, relevant dose differences included in the plan comparison were defined as follows. A relevant dosimetric improvement for an OAR reported in *Dosimetric Advantages Of Supplementary Static Apertures: Results* exceeds a dose level of 20% of prescribed dose with at least 3% difference between uncollimated PBS and PBS-with-apertures. The first criterion was not applied to the eye lens with a tolerance dose level of 5 GyRBE.

Craniopharyngioma cases were selected for a more detailed analysis in terms of some clinically relevant dosimetric parameters of the OARs. Six clinically delivered treatment plans were analyzed and compared. The prescribed treatment dose was 54 GyRBE.

### Data Workflow

Information about the field-specific apertures was passed within a DICOM RTIonplan from RayStation *via* the oncology information system (Mosaiq, Elekta, Stockholm/Sweden) to the TCS containing mounting position, material, label, thickness and a series of 2D points along the aperture contour. In RayStation and in the DICOM transfer a double set of identical apertures in the consecutive slots was coded by a single aperture of 6.5 cm thickness. Mosaiq provided an additional description for the beam shaping devices called “number of pieces”. This value was manually set in the course of the planning process. Mosaiq also facilitated the electronic interface to the MMS. It also acted as a record&verify system. In this frame, the field-specific apertures were tagged with a barcode label and scanned prior to field application.

### Quality Assurance

**Table 3** provides an overview of the implemented QA procedures. Generally, the QA program of a medical proton accelerator facility should be in line with the report of the AAPM task group 224 (15). That report does not cover the QA processes for PBS-with-apertures but for PBS combined with static field-shaping MLCs. Alternatively, the tolerance of the leaf position accuracy might be taken from TG 224, which is ±2 mm or ±1 mm if field patching or matching is performed. Aiming at an overall beam to target accuracy of less than 1 mm, we adopted the tighter limit of ±1 mm and defined a dedicated PBS-A QA-program as outlined in **Table 3**. In general, one has to test the co-incidence of three independent coordinate-systems: x-ray imaging, PBS and aperture. This means the origin of those need to be aligned at a common isocenter. The positioning of the aperture relative to the PBS field is impacted by the aperture manufacturing, but more importantly by the snout movement and aperture mounting mechanism. The co-incidence of aperture versus proton isocenter is tested on monthly basis with a tolerance of ±1 mm. The impact of this possible misalignment is evaluated for each individual treatment field in our TPS, as detailed in the next section. The daily QA procedures in our center (16) were not adapted. Furthermore, the outline of the milled opening of a fabricated aperture is visually compared against a printed hard copy from the TPS.

**Table 3:** Overview of quality assurance procedures. “MPE review” refers to the clinical release of a treatment plan by a certified medical physicist (“medical physics expert”).

Parameter	Warning level	Tolerance level	Test interval	Test device/comment
Co-incidence of coord. of X-ray field and PBS field	0.5 mm	1 mm	monthly	Lynx2D (EBT3 as alternative) with radio-opaque fiducial
Co-incidence of coord. of X-ray field and PBS field	–	1.5 mm	daily	spot positioning test Sun Nuclear DailyQA3
Co-incidence of aperture & PBS	–	1 mm	monthly	Lynx2D
Relative spot position	–	<5% of spots >1 mm	monthly	Lynx2D
	–	max 1.5 mm		
Outline of fabricated aperture	–	1 mm	field-specific	visual test with print-out
Dose plane of fields: γ -test	γ < 1	γ <1	field-specific	DigiPhant;
	for <95%	for <90%		global γ 3%/2 mm, 10% dose threshold
Number of apertures correct	–	pass/fail	field-specific	Python script; MPE review

EBT3 is a radiochromic film of the vendor Ashland. Lynx2D is a scintillation screen with electronic readout (IBA dosimetry). **“**coord.**”** is used as abbreviation for **“**coordinates**”**. The Python script runs within the RayStation treatment planning system.

### Robustness Analysis

As shown in previous works [see, e.g., Refs. (17, 18)] the geometrical expansion of a structure with a margin could fail to prevent underdosage of the target volume and overdosage of organs-at-risk in particle therapy. Consequently, a robustness evaluation based on perturbed dose scenarios was established as part of our treatment planning QA adhering to the concept of Ref. (18). Using the built-in function of RayStation to compute a perturbed dose distribution, a simultaneous set-up shift in all cardinal directions with the same sign combined with a rescaling of the mass density, was performed for all treatment plans. Considering a shift and a rescaling with both signs, this yielded four perturbed scenarios. The parameters of the perturbations were chosen to get a confidence level of 90%.

If apertures are used for field shaping, a systematic offset of spot positions and a systematic offset of the aperture contribute to the set-up error. The spot position offset and the aperture offset could have different signs and different values as outlined in the previous section. This kind of perturbation scenario is not covered by the built-in perturbation tool of RayStation. In our customized Python script four copies of the clinical plan were created which would serve as perturbed scenarios of the nominal plan. In each copy the coordinates of the aperture contour were shifted by ±1 mm independently in each lateral direction. The dosimetrist selected the worst case scenario of the four so-established perturbed scenarios of misaligned apertures, which is in turn were used as a starting scenario of the conventional perturbation analysis.

## Dosimetric Advantages of Supplementary Static Apertures: Results

**Figure 2:**
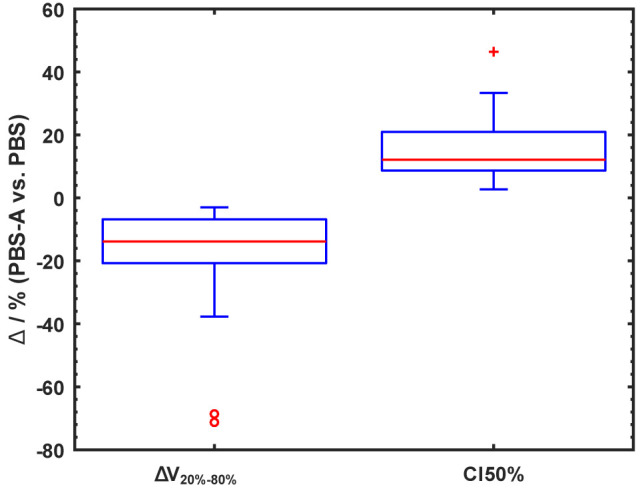
Percentage difference of ΔV_20-80%_ and CI 50% between PBS plans with and without collimating apertures visualized in a box-whisker plot. ΔV_20-80%_, volume between 20 and 80% isodose lines; CI 50%, conformity index of 50% isodose line; PBS, uncollimated pencil beam scanning; PBS-A, pencil beam scanning with apertures. The boxes (whisker) indicate the 25th and 75th percentiles (1.5 times interquartile range). Data points outside the three times (1.5 and three times) the interquartile range are indicated by open circles (plus symbol). Median values are indicated by red lines.

The overall reduction of the dose-volume between 80 and 20% isodose lines (ΔV_20-80%_) is depicted in **Figure 2**. A clear dose reduction of the volume around the target volume is visible. The addition of collimated apertures reduces ΔV_20-80%_ on average by 17.6% (median 13.8%). For the full cohort of 31 cases the mean brain dose could be reduced on average by 1.2 GyRBE through apertures. The CI of the 50% isodose line increased on average by 15.3% (median 12.1%) when PBS-with-apertures was used. **Table 4** shows the dosimetric improvements per tumor entity. This table reflects the variation of the dosimetric impact of auxiliary apertures among cases.

**Table 4:** Percentage difference in ΔV_20-80%_ and CI 50% for PBS-with-aperture plans compared with uncollimated PBS plans.

Tumor entity	Vol. 20–80%	CI-50%
Orbital tumors	−27.0%	18.0%
Craniopharyngioma	−13.6%	13.6%
Ependymoma	−16.0%	8.0%
Astrocytoma	−3.3%	7.8%
Retreatment	−30.4%	21.0%
Other brain tumors	−7.7%	17.3%

**Figure 3:**
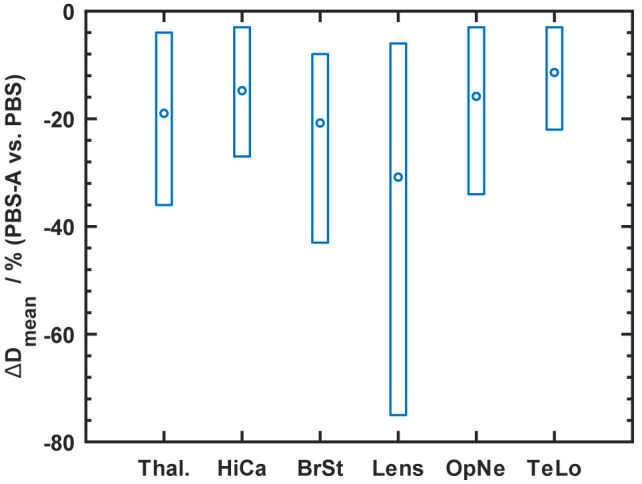
Summary of the mean dose (D_mean_) reduction for OARs using PBS-with-apertures (“PBS-A”) indicated by circles. The boxes show the full range of achieved reductions. The shown structures are Thal, thalamus; Hica, hippocampus; BrSt, brainstem; OpNe, eye lens, optical nerve; TeLo, temporal lobe.

The most noticeable improvement regarding dose sparing is a dose reduction of the hippocampi, which varied between 1.6 and 4.7 GyRBE with a mean value of 2.9 GyRBE. The level of the mean dose for the hippocampi of the considered cases was on average 20.3 GyRBE. **Figure 3** provides an overview of the dose reduction facilitated with field-specific apertures for selected OARs. In case of bi-lateral structures, the value refers to the structure receiving the higher dose.

**Figure 4:**
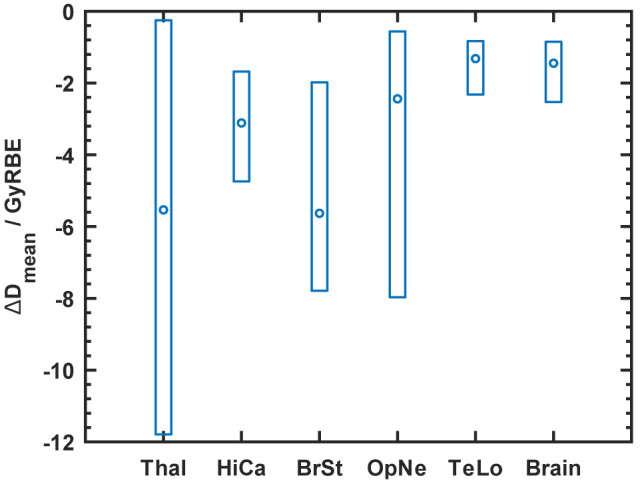
Dose reduction to organs-at-risk (OAR) for craniopharyngioma cases comparing plans with aperture (“PBS-A”) with uncollimated plans (“PBS”). Percentage difference of D_mean_ for organs-at-risk between the plans with and without apertures. The boxes show the full range of achieved reductions. The shown structures are Thal, thalamus; HiCa, hippocampus; BrSt, brainstem; OpNe, optical nerve; TeLo, temporal lobe; and brain.

**Figure 5:**
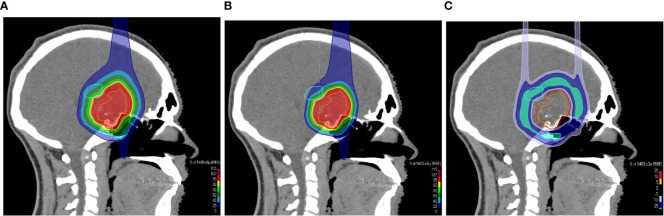
Sagittal CT slices overlaid with a colorwash representation of the dose distribution of the uncollimated plan **(A)** and the treatment plan with apertures **(B)** for the treatment of a suprasellar craniopharyngioma. The screenshots of the treatment planning in RayStation show the dose sparing of the left thalamus, which is indicated by the light blue contour. **(C)** shows the dose difference between the plans.

The target volumes of the six craniopharyngioma cases are located near the most important OARs like brainstem, optic nerves, chiasma and hippocampi. Usually target volumes overlap with OARs. For this reason almost no difference was observed for the dose maximum (D_max_) or the dose receiving 1% of a considered volume (D_1_). Therefore, the average dose of OARs (D_mean_) was compared even for serial-type OARs. An absolute dose difference was calculated and summarized in **Figure 4**. The biggest benefit of using PBS-with-apertures was achieved for thalamus, brainstem, and hippocampus reducing D_mean_ by 5.5, 5.6 and 3.1 GyREBE, respectively. The rather large variation in dose reduction for the thalamus could be explained by the varying proximity to the target volumes. **Figure 5** shows the exemplary dose distribution of a case, which benefited most from the use of apertures in terms of sparing of both thalami.

**Figure 6:**
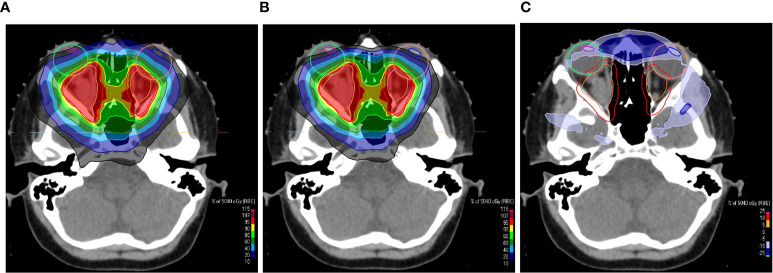
Transversal CT slices overlaid with a colorwash representation of the dose distribution of the uncollimated plan **(A)** and the plan with apertures **(B)** for the treatment of an opticus glioma. The screenshots of the treatment planning in RayStation show the dose sparing of the eye lenses, which are indicated by the blue and magenta contours. **(C)** shows the dose difference between the plans. The beams eye view of one of the treatment fields is shown in **Figure 1**.

For two cases the PBS-with-aperture technique facilitated a qualitative improvement of OAR sparing by reducing the mean dose for the eye lens below 5 GyRBE. This would not have been achieved with open PBS fields. For these two cases OAR sparing had the highest priority. Consequently, the PTV coverage was compromised in the uncollimated plans. **Figure 6** provides an example of the dose distribution for one of these cases.

**Figure 7:**
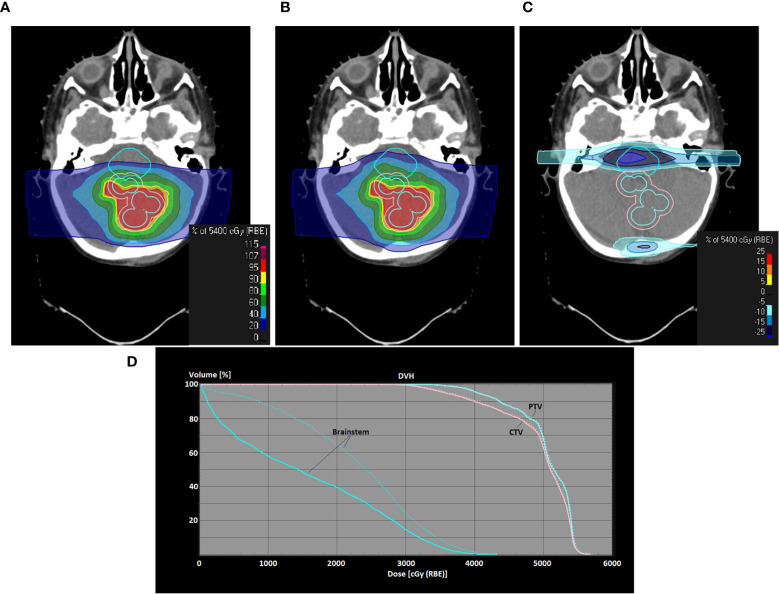
Use of PBS-with-apertures for one of the retreatment cases (metastasis of an atypical teratoid rhabdoid tumor in the fossa posterior). Screenshots of treatment planning in RayStation are shown. Dose distributions in a transversal plane are shown as colorwash **(A–C)** PBS-with-apertures, uncollimated PBS plan, dose difference between PBS-with-apertures and uncollimated PBS plans; **(D)** dose volume histogram showing the difference for the brainstem and the target volumes between uncollimated (dashed line) and collimated (solid line) plans. PTV/CTV/brainstem is contoured with an orange/light blue/cyan line.

Two retreatment cases were planned with a similar approach. Here, the maximum dose to the brainstem could be reduced. **Figure 7** provides an example. The primary goal was the sparing of the brainstem. The dose gradient at the interface between PTV and brainstem is squeezed (**Figure 7A**) compared to the uncollimated plan (**Figure 7B**). The OAR constraints could already be met with uncollimated plans for the other 27 cases. In those cases the apertures facilitate an extra sparing of normal tissue.

## Discussion

### Clinical Benefits of Treatment Plans Using Apertures

The benefits of PBS-with-apertures could be assessed by comparing with previous studies, which used a similar technique (2, 19–21). Furthermore, the dosimetric improvements were compared to *in silico* studies of PBS combined with dynamic collimation (8, 14). As pointed out in Ref. (14), that technique is supposed to be superior to PBS with static apertures in terms of conformality and served, thus, as a best case of proton PBS with extra beam shaping hardware. To the best of our knowledge, the current study is together with Ref. (19) the only one about patients treated with PBS in conjunction with supplementary collimators. In this regard it’s the first study evaluating intracranial and orbital tumors. Furthermore, the patient cohort is clearly larger as in the previous studies mentioned above.

The current study identified a benefit for the hippocampi. For cases with a relevant sparing of the hippocampi the dose could be reduced on average by about 3 GyRBE (**Figure 4**). References (22, 23) stressed the clinical relevance of dosimetric improvements of this size for the hippocampi. Therefore, optimal sparing of the hippocampi is increasingly considered an important aim in treatment planning. For instance, the SIOP PNET 5 trial (ClinicalTrials.gov identifier: NCT02066220) and the SIOP Ependymoma Program II (ClinicalTrials.gov Identifier: NCT02265770) require contouring and dose reporting of the hippocampi. Clinical data about side effects are sparse and recent studies regarding neurocognitive impairment appear to be inconsistent (24, 25). The dose reductions achieved in the current paper are expected to reduce the probability of neurocognitive impairment (24, 26), which is currently hard to quantify with normal-tissue complication probability (NTCP)-models (23, 25, 27).

This study found a mean reduction of ΔV_20-80%_ by 13.6%. Reference (14) reported about a 5.2% reduction of the mean dose of a 10 mm thick ring surrounding the PTV. That study also pointed out that the mean dose to the 10 mm ring could be reduced with dynamic collimation on average by 13.7%. The average reduction of mean dose to the healthy brain with energy-layer specific dynamic collimation was on average 25% in Ref. (8).

As for almost all uncollimated plans the dose to the OARs was already well below the tolerance level, a benefit of supplementary apertures for a risk-adapted adjustment of the high-dose volume was reported only in two out of 31 cases. Of course, tabulated OAR tolerances cannot be applied to the retreatment cases, which clearly benefit from a general reduction of the dose (**Figure 7**). In two retreatment cases only PBS-with-apertures achieved an acceptable brainstem dose. Furthermore, the expected reduction of side effects also includes secondary malignancies. For instance, three cases of the considered eye treatments were retinoblastoma. Generally, we expect the biggest impact on normal-tissue complication, if the spatial extent of the involved structures and distances between them are on the scale of the dose fall-off, which is about 1 cm (4). The advantage at small spatial distances makes PBS-with-apertures a viable technique for localizations in the skull or for pediatric patients. The cochleae and the pituitary gland are examples for small-sized structures, which potentially develop less late effects through dose sparing with collimators. As pointed out in Ref. (28), there is evidence for a correlation of late end-points, e.g. hearing loss and endocrine dysfunctions, with absorbed dose in proton therapy. However, there are still uncertainties in the radiobiological models, including a possible influence of a heterogeneous dose distribution in the OARs. The clinical impact of the dose reduction of OARs by several GyRBE, which could be achieved by the insertion of apertures, was difficult to predict. Thus, future studies should seek to improve the NTCP-models, considering both the proton beam radiation quality (29, 30) and the irradiation of pediatric patients (31).

The outcome of the current dosimetric study could be regarded as a minimum achievement, which could be expected from field-specific apertures, because a horizontal beam-line was used. Reference (20) performed a similar comparison for two cases with a gantry-mounted nozzle of the same type as used here. Reductions of the mean dose of the cochlea of about 50% were reported. Dose sparing of abdominal organs was on the order of 20%. These relative improvements are clearly larger than the ones reported in this study indicating the synergy of PBS, field-specific apertures and a gantry. One may emphasize in this context the relevance of the choice of the beam ports in proton therapy, especially if an OAR is in the vicinity of the target volume. The strong distal dose gradient would provide an effective means to separate the high dose volume from the nearby OAR. However, the position of the distal dose fall-off in the patient is subject to uncertainties stemming from the range uncertainty of the proton machine (≈1 mm) and the stopping power estimation (≈3.5%). Moreover, the distal edge of proton fields features larger linear-energy transfers which are presumably associated with an elevated relative biological effectiveness [see, e.g., Refs. (32, 33)]. This is especially a concern for pediatric cases (34, 35). As a consequence, the treatment planning in our proton center seeks to avoid the ranging out of fields on common OAR tissue for more than one third of the fraction dose, as discussed in (35). This limitation motivates the importance for a sharp transveral dose fall-off and, thus, additional collimation of PBS fields. We further conceive the blocking of the myelon, e.g. for infratentorial targets, and the blocking of vertebrae to avoid spine deformation for pediatric patients as possible clinical applications of PBS-with-apertures. This was not covered in the current study due to a quite limited cohort size and the lack of a gantry. The restriction to a horizontal treatment head is the main reason why extracranial targets have not been treated with PBS-with-apertures in our center.

The improvements of the dose distribution, which have been identified in this study, could be limited by the arrangement of beam modifiers. As shown in previous studies (4, 6) the reversed order of aperture and range shifter, i.e. the aperture downstream of the range shifter, features a steeper transversal dose gradient for divergent scanning fields. The usage of a corresponding snout would allow for an improved CI 50% and even less dose burden to healthy tissue. This comes at the expense of a limited effect of the fluence modulation on dose shaping due to broadened PBS spots. Albeit an easy mechanical modification, the current work only considered the nominal configuration of the Snout180 adhering to the intended use of the proton machine according to its vendor. For more information about the fundamentals of trimming PBS spots with a metal collimator, it is referred to Ref. (7).

### Out-of-Field Dose and Radiation Protection Issues

One may note that the RayStation MC dose engine does not simulate secondary neutral particles. Thus, the out-field-dose, i.e. scattered dose far away from the target volume, is not covered in the presented dose distributions. In a MC study (36) it was found that the inclusion of a brass aperture causes a higher neutron fluence than the nominal PBS field configuration without aperture. Thus, the neutron contribution to the out-of-field dose equivalence increases when supplementing PBS with brass apertures. The same study assessed, however, that the use of a brass aperture leads to a reduction of the overall out-of-field dose. A similar conclusion was drawn in Ref. (37) in which the impact of a dynamic collimator was investigated. Using a thick graphite range shifter and a pair of nickel trimmers, the secondary neutron ambient dose equivalence was typically 70% larger as compared to the uncollimated field configuration. According to the MC simulation study of Ref. (38) hadronic interactions in nickel cause about 8% less neutron fluence/dose than in brass. As the impact of secondary neutrons was mainly evaluated in simulations, which depend on the models for neutron interactions (39), more experimental data are needed. Furthermore, this study did not investigate the effect of an increased linear-energy transfer (LET) of protons scattered from the aperture edge. Ueno et al. found a small increase of the dose-averaged LET when field-specific collimators were added (40).

The radiation protection of personnel concerns mainly the manual procedure of unmounting the apertures, which is performed by RTTs or physicists. Reference (41) showed with gamma-ray spectrometry and with MC simulations that the short-term radioactivation is dominated by the isotopes ^63^Zn, and ^60,61,62^Cu. The delayed, secondary radiation from these nuclei is a minor radiation protection concern, because the equivalent dose rate induced by the emitted photons is on the level of the natural background radiation. The long-lived isotopes, which are most relevant for storage and disposal, are ^57,58^Co, ^65^Zn, and ^54^Mn (41). Assuming a dose prescription of 50 GyRBE, the accumulated activity after 30 fractions of long-lived isotopes is at the level of the exemption limit for recycling, especially for ^65^Zn. The need for a long-term storage prior to clearance depends on the irradiation volume of the aperture and the energy spectrum of the incident protons (42). Thus, individual measurements of activity or equivalent dose rate are necessary and a storage room for decay has to be available (43).

## Summary

The addition of field-specific apertures to pencil-beam scanning treatment fields was successfully introduced at a proton therapy center which is based on commercial equipment. Pencil-beam scanning with custom-fabricated apertures has been clinically released in a treatment room with a horizontal beam line. The extra hardware effort mainly concerns a computer controlled milling machine and space for the decay of radioactivated apertures. The additional effort for quality assurance is moderate and can be derived from established procedures. In treatment planning, supplementary apertures were combined with robust optimization and integrated into the robustness evaluation.

Selected patients were treated with supplementary apertures. The *in silico* study indicated dosimetric advantages by comparison with treatment plans using uncollimated fields. The biggest dosimetric advantage was assessed for organs at risk in the vicinity of the high-dose, e.g. the hippocampus or the thalamus. Furthermore, the conformity index improved by typically 10–20% which is related to an overall decreased dose burden to healthy brain tissue. Apertures can facilitate the sparing of an organ-at-risk while keeping the dose coverage of the target, which was achieved in 4/31 cases.

## Data Availability Statement

The original contributions presented in the study are included in the article/supplementary material. Further inquiries can be directed to the corresponding author.

## Ethics Statement

Ethical review and approval was not required for the study on human participants in accordance with the local legislation and institutional requirements. Written informed consent to participate in this study was provided by the participants’ legal guardian/next of kin.

## Author Contributions

CB and SP wrote the manuscript. CB performed the clinical commissioning tests. SP evaluated the dose metrics and compiled the statistics. DG, SP, P-HK, DA, and BT did the treatment planning of the patients. JW took care of the extension of the QA program. CN and KK assessed the radioprotection aspects. All authors contributed to the article and approved the submitted version.

## Funding

The authors are thankful to the HARMONIC project. The HARMONIC project (Health effects of cArdiac uoRoscopy and MOderN radIotherapy in paediatriCs) has received funding from the Euratom research and training programme 2014-2018 under grant agreement No 847707.

## Conflict of Interest

The authors declare that the research was conducted in the absence of any commercial or financial relationships that could be construed as a potential conflict of interest.
